# Link-Level Functional Connectivity Neuroalterations in Autism Spectrum Disorder: A Developmental Resting-State fMRI Study

**DOI:** 10.3390/diagnostics9010032

**Published:** 2019-03-21

**Authors:** Lluis Borràs-Ferrís, Úrsula Pérez-Ramírez, David Moratal

**Affiliations:** Center for Biomaterials and Tissue Engineering, Universitat Politècnica de València, 46022 Valencia, Spain; lluisb3@gmail.com (L.B.-F.); ursulaperezr@gmail.com (Ú.P.-R.)

**Keywords:** autism, brain functional connectivity, default mode network, full correlation analysis, partial correlation analysis, region of interest analysis, resting-state functional MRI

## Abstract

Autism spectrum disorder (ASD) is a neurological and developmental disorder whose late diagnosis is based on subjective tests. In seeking for earlier diagnosis, we aimed to find objective biomarkers via analysis of resting-state functional MRI (rs-fMRI) images obtained from the Autism Brain Image Data Exchange (ABIDE) database. Thus, we estimated brain functional connectivity (FC) between pairs of regions as the statistical dependence between their neural-related blood-oxygen-level-dependent (BOLD) signals. We compared FC of individuals with ASD and healthy controls, matched by age and intelligence quotient (IQ), and split into three age groups (50 children, 98 adolescents, and 32 adults), from a developmental perspective. After estimating the correlation, we observed hypoconnectivities in children and adolescents with ASD between regions belonging to the default mode network (DMN). Concretely, in children, FC decreased between the left middle temporal gyrus and right frontal pole (*p* = 0.0080), and between the left orbitofrontal cortex and right superior frontal gyrus (*p* = 0.0144). In adolescents, this decrease was observed between bilateral postcentral gyri (*p* = 0.0012), and between the right precuneus and right middle temporal gyrus (*p* = 0.0236). These results help to gain a better understanding of the involved regions on autism and its connection with the affected superior cognitive brain functions.

## 1. Introduction

Autism spectrum disorder (ASD) is a neurodevelopmental condition that starts in childhood and lasts a lifetime. ASD introduces three fundamental characteristics: qualitative disruption of relationships, alterations in the communication and speaking skills, and a lack of mental and behavioral flexibilities. This suggests that these deficits observed in patients with ASD are the result of a deficient executive function that includes problems in working memory, inhibition, mental flexibility, and planning [1]. Worldwide, the prevalence of ASD is 1 in 100 children [2]. Unfortunately, no treatment can cure this disorder and, due to the heterogeneity in phenotype, personalized therapy is necessary.

Nowadays, the diagnosis of ASD consists of a combination of clinical and psychological tests that aim to detect the associated symptoms of this disorder using the Diagnostic and Statistical Manual of Mental Disorders (DSM) [3]. Consequently, the diagnosis is focused on observable symptoms and not on early signs. For this reason, it is subjective and late, since the symptoms usually appear from the age of three years old. Therefore, it is of utmost importance to provide biomarkers to characterize the brain’s functional alterations using functional magnetic resonance imaging (fMRI) images of the brain [4]. Commonly, MRI images are being used in this field because they are a powerful non-invasive tool for studying the developmental trajectory of the brain. Quantitative MRI biomarkers promise to achieve early diagnosis of this disorder which would increase the effectiveness of therapies to improve the behavior and communication of autistic people [5].

The analysis of low-frequency oscillations in the blood-oxygen-level-dependent (BOLD) signal, extracted from fMRI images, indirectly allows calculating neuronal activity. Functional connectivity (FC) is defined as the statistical dependence between BOLD signals. FC shows how the regions are organized and interrelated, and reveals which regions communicate with other regions to serve specific functions [6]. To diagnose ASD, it is important to acquire fMRI images in resting state (rs-fMRI) because one of the most important brain networks with anomalies in FC due to ASD—the default mode network (DMN)—it is activated in rest [7].

Several research studies show discrepancies in brain FC in the DMN when individuals with ASD are being compared with control subjects: hyperconnectivity (children [8,9] and children together with adolescents [10,11]) or hypoconnectivity (children and adolescents [12]; children, adolescents, and adults [13], adolescents [14]; adolescents and adults [15]; and adults [16,17]). The theories of hypoconnectivity explain a reduction in the interregional neuronal connections, whereas the hyperconnectivity studies an increment of neuronal connections between some brain regions [1]. Among the possible causes of these contradictory results are (1) changes in brain development because of a wide age range [18], (2) a different methodology of analysis and different fMRI acquisition parameters [19], and (3) ASD heterogeneity [1].

Our purpose was the statistical analysis of the alterations in brain FC inside the DMN and other brain regions directly implicated between autists and healthy controls. The subjects were divided into different age ranges (children, adolescents, and adults), to study autism from a developmental point of view, performing a correlation analysis between brain regions from rs-fMRI images. With the separation in age ranges we aim to give a response to the controversy of hyper- and hypoconnectivities between the same brain networks in previous studies where subjects were not divided into age groups, despite ASD being a neurodevelopmental condition. In contrast to seed-based correlation analyses (SCA) [20] and independent component analysis (ICA) [21] that are focused on functionally connected networks with connectivity weights, ROI-based analyses [22] facilitate the biological interpretation of the results because the ROIs are anatomical brain regions with a known function.

## 2. Materials and Methods

### 2.1. Subjects and Image Acquisition

The rs-fMRI images of ASD and control subjects were obtained from the public and multicenter Autism Brain Image Data Exchange (ABIDE) [23] database. This database offers preprocessed rs-fMRI images and demographical information of 1112 subjects from 16 universities (539 ASD individuals and 573 typical controls) [24]. In this study, only right-handed men were included because they are the majority gender in the database (>75%), and from ASD individuals only the autists. Three age ranges were established: children (<12 years old), adolescents (12–18 years old), and adults (>18 years old), to take into account the development, matching in each age range the controls and the autists by age (±2 years old in children and adolescents, and ±5 years old in adults) and intelligence quotient (IQ) (±10 points). The characteristics of the subjects that belonged to this study are shown in Table 1. Regarding the children, two groups were considered: (1) children whose images were acquired in different universities and (2) children solely scanned in the New York Langone Medical Center (NYU), to study the importance of the homogeneity in rs-fMRI acquisition. We selected the the NYU dataset rather than the University of California, Los Angeles (UCLA) or the University of Michigan (UM) datasets because it contained more subjects.

### 2.2. Preprocessing

**Figure 1:**
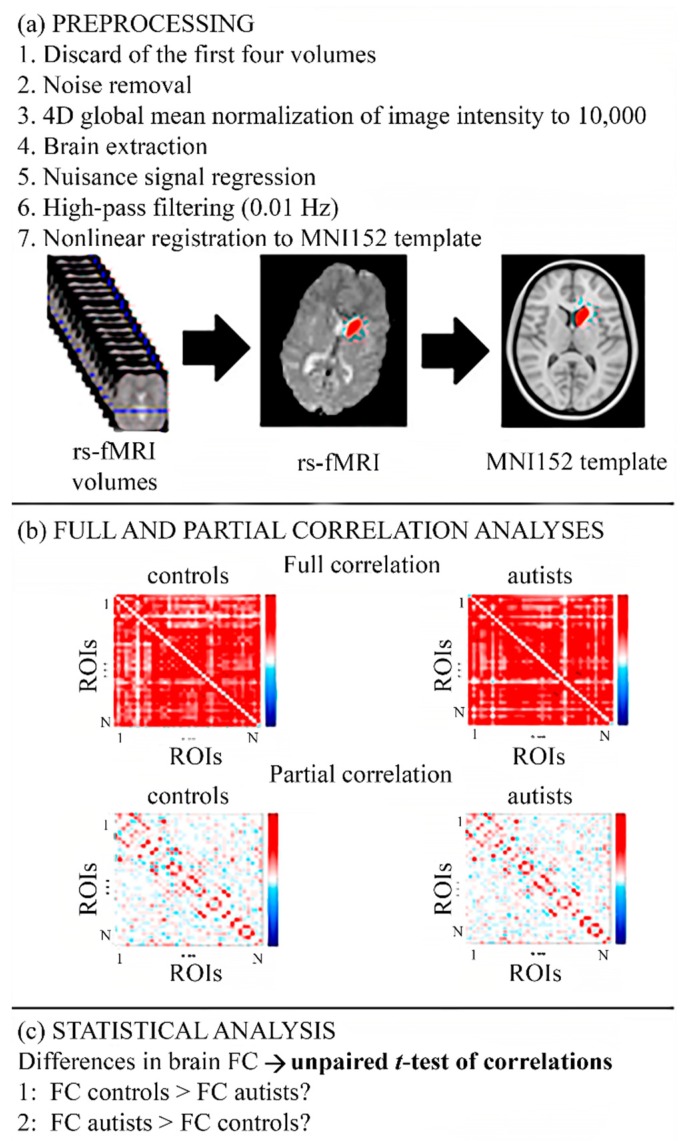
Flowchart of the methodology to compare functional connectivity in controls and autists. (**a**) Preprocessing steps for the rs-fMRI images. (**b**) Full and partial correlation analyses. Correlation indicates the amount of statistical dependence between two anatomical regions. Full correlation measures the amount of FC—statistical dependence—between two directly or indirectly connected regions, whereas the partial correlation focuses on direct connections. (**c**) Statistical analysis with two contrasts: (1) FC between two regions is higher in controls than in autists, (2) FC between a pair of regions is greater in autists than in controls. FC: functional connectivity; ROIs: regions of interest; rs-fMRI: resting-state functional magnetic resonance imaging.

The ABIDE team preprocessed the rs-fMRI images with Connectome Computation System (CCS) [25]. The main steps (Figure 1a) were (1) discarding of the first four brain volumes; (2) noise removal—brain volumes with extreme intensity, slice timing, and motion correction; (3) brain extraction; (4) 4D global mean normalization of image intensity with a value of 10,000; (5) nuisance signal regression (Friston’s 24-parameter motion curves, mean time series of white matter and cerebrospinal fluid, then linear and quadratic trends); (6) high pass filtering (0.01 Hz) to remove the continuous component and (7) nonlinear registration to MNI152 (Montreal Neurological Institute, McGill University, Montreal, Canada) template.

### 2.3. rs-fMRI Time Series Extraction

First, 38 anatomical regions of interest (ROIs) were selected (19 ROIs in each brain hemisphere, Table 2) belonging to the core DMN or with a high probability to be affected in autism [18]. All the ROIs are included in the Harvard–Oxford brain atlas [26]. The ABIDE team publicly shared the mean temporal signals from each ROI; concretely, for each voxel of the ROI, the time series is extracted and then the mean of all these signals is computed.

### 2.4. Full and Partial Correlation Analyses

A correlation shows the power and the direction in which two or more variables are linearly associated. In this study, the amount of FC present between two brain regions is shown. Full correlation measures the amount of FC between two signals directly connected with each other or indirectly through other regions, and it is calculated by Equation (1):

(1) $$corr_{XY}=\frac{cov_{XY}}{S_{X}S_{Y}},$$

where *X* and *Y* are BOLD temporal signals, $cov_{XY}$ is the covariance between *X* and *Y*, and *S* is the standard deviation of the temporal signals. The main unit of full correlation is the *ρ* of Pearson with values in the range of [−1, 1], which is recommended to transform to Fisher’s *Z* [27].

Partial correlation is calculated similarly as full correlation, but the inverse covariance matrix is used. This correlation has the objective of estimating only the direct functional connections between pairs of regions, meaning between regions anatomically connected to each other. Pursuing this aim, the correlations between the pair of signals under study are calculated once the variance of the rest of the signals is linearly removed [27]. In this study, L2-regularized partial correlation was used with Tikhonov regularization. This regularization helps to get a stable approximation of the exact solution in the case of a wrong approach of the equations due to noise [28]. The correlation value between each pair of regions is shown in a matrix of correlation with size N × N, with N being the number of regions under study (Figure 1b).

The statistical correlation analysis was performed with FSLNets v0.6, part of the neuroimaging tool FSL (FMRIB Software Library, Oxford Centre for Functional MRI of the Brain, Oxford, UK) [29,30] and additional scripts in MATLAB R2016a (The MathWorks, Inc., Natick, MA, USA).

### 2.5. Statistical Analysis

The characteristics of the study were modeled using the general linear model (GLM) [31] written in Equation (2):

(2) Y=X·B+ε,

where *Y* is the correlation matrix obtained from the rs-fMRI signals, *X* is the design matrix that classifies controls or autists, and models the age and the IQ (both in relation to the mean of all the individuals), and the acquisition center, *B*, is the matrix with the regression parameters to get, and *ε* is the noise, with mean zero and standard deviation of 1.

The null hypothesis covered the fact that the full or partial correlation between two regions did not differ between controls and autists. Two alternative hypotheses or contrasts were defined (Figure 1c): (c1) FC in controls was greater than in autists and (c2) FC in autistic individuals was greater than in controls. The values of *p* were corrected with family-wise error rate (FWER) by 5000 permutations [32]. The threshold for statistical significance was fixed as 0.025 because there were two contrasts.

## 3. Results

The results were grouped by age range: children, adolescents, and adults. As the significant results were only found in full correlation, and not in partial correlation, it seems that there were indirect connections between regions.

### 3.1. Functional Connectivity in Children

**Figure 2:**
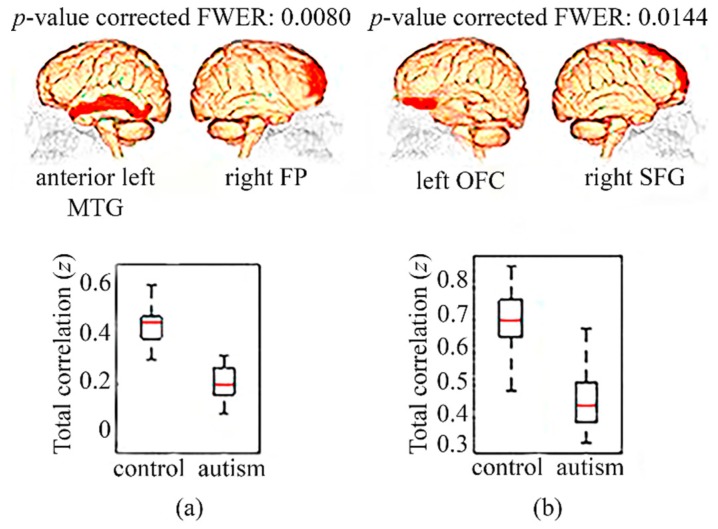
Hypoconnectivities in children (<12 years old) with autism compared to controls, from the NYU center. (**a**) Boxplot showing full correlation values between anterior left MTG and right FP in controls and autists; (**b**) Boxplot showing the full correlation values present in controls and autists between the regions left OFC and right SFG. FP: frontal pole; MTG: middle temporal gyrus; NYU: New York Langone Medical Center; OFC: orbitofrontal cortex; SFG: superior frontal gyrus.

In the analysis, taking into account only the subjects from NYU, two pairs of regions showed significant results in full correlation (Figure 2). Concretely, there was hypoconnectivity in the autistic children with respect to the controls in the following pairs of regions: anterior middle temporal gyrus (aMTG) in the left hemisphere and the right frontal pole (FP) (Figure 2a); and between the orbitofrontal cortex (OFC) in the left hemisphere and the right superior frontal gyrus (SFG) (Figure 2b).

### 3.2. Functional Connectivity in Adolescents

**Figure 3:**
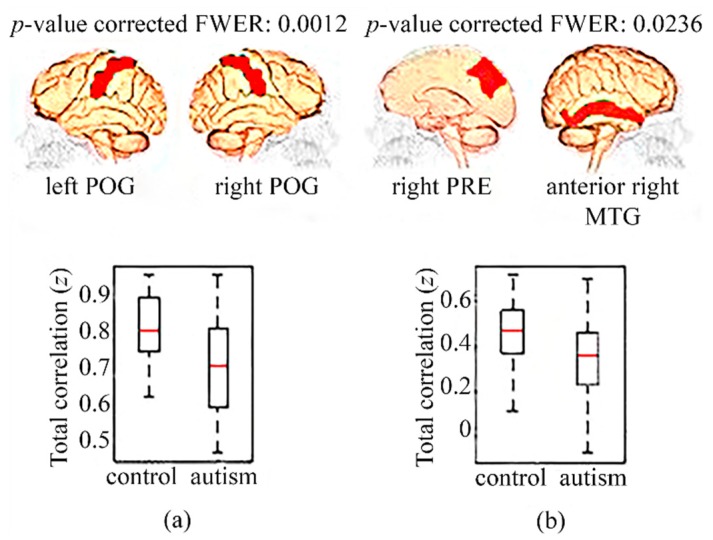
Hypoconnectivities in adolescents (12–18 years old) with autism relative to controls in a multicenter study. (**a**) Boxplot showing full correlation Fisher’s z values between left and right POG in controls and autists; (**b**) Boxplot showing the full correlation Fisher’s z values between the right PRE and anterior right MTG present in controls and autists. MTG: middle temporal gyrus; POG: postcentral gyrus; PRE: precuneus.

Figure 3 shows the significant differences in full correlation in adolescents. There was hypoconnectivity in autists with respect to controls between these brain regions: postcentral gyrus (POG) in both hemispheres (Figure 3a) and between the precuneus (PRE) of the right hemisphere and the anterior MTG of the right hemisphere (Figure 3b).

### 3.3. Functional Connectivity in Adults

In adults, no significant statistical differences in FC were found in the multicenter analysis.

## 4. Discussion

By means of the estimation of full correlation, significant hypoconnectivities in autistic children from the NYU center have been found in relation to children without autism from the same center (<12 years old) in regions of the DMN, specifically between the anterior left MTG and the right FP, and between the left OFC and the right SFG. Moreover, several decrements of FC in the autistic adolescents (12–18 years old) from the multicenter analysis were also present in the DMN, concretely between both hemispheres of the POG and between PRE in the right hemisphere and the right anterior MTG. On the contrary, there were no significant changes in FC in adults (>18 years old). These six regions with a hypoconnectivity in autists relative to controls are directly associated with superior cognitive brain functions, including language, integration of sensorial information, deductive reasoning, and processing functions of memory as a working memory. In addition, some of these regions are involved in functions directly connected with the behavior and the learning of individuals [33,34,35,36,37,38]. Therefore, hypoconnectivity in these brain areas might be an indicator of a deficit of the corresponding brain functions [1].

With respect to the studies only centered on children, there is controversy because the studies of Lynch et al. [8] and Uddin et al. [9], both with an age range of 7 to 12 years old, observed hyperconnectivity of the autism patients’ in relation to the controls inside the DMN. However, the results of the study of Weng et al. (13–18 years old) [14], focused only on adolescents, are consistent with the results of this study, showing a hypoconnectivity inside the DMN. The studies focused only on children and adolescents have led to controversies as well. On the one hand, the studies of Rudie et al. (13 years on average) [12] and Assaf et al. (10–23 years old) [13] are consistent with this study: hypoconnectivity in autists inside the DMN. On the other hand, the studies of Di Martino et al. (7–13 years old) [10] and Washington et al. (6–17 years old), using independent component analysis (ICA) [11], obtained a hyperconnectivity inside the DMN of the autists. We did not get statistical significance in the study focused on adults, but in the studies of Kennedy et al. (15–52 years old) [15], Mueller et al. (33 years old) [16], and von dem Hagen et al. (19–40 years old) [17] they found a hypoconnectivity inside the DMN.

It is important to highlight that only the children from NYU center and the adolescents provided significant results, but not the adults and children from the multicenter study. On the one hand, comparing the multi- and unicenter study in children, it seems that the homogeneity in the acquisition was more important than having more heterogeneity in the individuals from other centers. On the other hand, the group of adolescents came from different centers, but the number of individuals was double that in the children group, showing that a high number of individuals is crucial to overcoming the heterogeneity limitations of a multicenter study, despite a good design. In the multicenter analysis of adults, the total number of individuals was the least of all the studies.

In the present study, the individuals have been divided into three age ranges, due to the importance of studying autism from a developmental point of view to account for anatomical and functional characteristics of each period [18]. Due to brain plasticity, connectivity differs in children, adolescents, and adults [1]. IQ was also considered to match the subjects.

One of the limitations of this study is a small sample size because the individuals were grouped by age ranges, a problem that the ABIDE II initiative is tackling [39]. In addition, the fact that a medium group from one center obtained significant results confirms the importance of data homogenization or good controls in the design matrix. The models used in this study are a key factor to influence the results. To create the statistical model, the principal methods of analysis are ICA, a method that expects to find a linear combination of non-Gaussian data [20]; SCA, to measure the FC maps from the correlation of the seed signal with all the brain signals [21]; and ROI analysis, a method to measure FC between all pairs of ROIs [22]. Compared to ICA and SCA, anatomical ROI analysis has been chosen for simple biological interpretation, because each ROI belongs to a specific and localized area of a brain atlas.

## 5. Conclusions

Changes in brain FC were studied between control and autists individuals, divided into three age ranges (children, adolescents, and adults), taking into account the importance of studying autism from a developmental point of view, and matching also by IQ, to minimize the heterogeneity of autism. The study of brain FC between pairs of ROIs was performed by a statistical analysis of full and partial correlation. The results show hypoconnectivities in the autistic individuals relative to matched controls, between two pairs of regions in the children group and another two pairs of regions in the group of adolescents, all of them inside the DMN. These regions are directly involved in cognitive, behavioral, and learning functions. These results were obtained only in the full correlation analysis, showing that these regions are connected through other regions, meaning that they are not directly connected. In the adult group, no significant differences were found.

## Figures and Tables

**Table 1:** Individuals from the public database ABIDE (Autism Brain Image Data Exchange) used in this study. CMU: Carnegie Mellon University; KKI: Kennedy Krieger Institute; Leuven: University of Leuven; NYU: New York Langone Medical Center; Pitt: University of Pittsburgh School of Medicine; Trinity: Trinity Center for Health Sciences; UCLA: University of California, Los Angeles; UM: University of Michigan.

Age Range	Number of Individuals (Controls/Autists)	Average Age ± Standard Deviation	Universities or Research Centers
Children (<12 years old)	25/25 11/11	10.63 ± 0.86 10.47 ± 0.86	NYU, UCLA, UM, NYU
Adolescents (12–18 years old)	49/49	14.35 ± 1.77	Leuven, NYU, Pitt, Trinity, UCLA, UM
Adults (>18 years old)	16/16	23.41 ± 3.76	CMU, Leuven, NYU, Pitt

**Table 2:** Thirty-eight anatomical regions selected for the study (19 regions in each brain hemisphere), part of the default mode network (DMN) and included in the Harvard–Oxford brain atlas.

19 Anatomical ROIs in Each Cerebral Hemisphere		
Amygdala (AMG)	Inferior frontal gyrus “pars triangularis” (IFGpt)	Hippocampus (HIP)
Insular cortex (INC)	Middle frontal gyrus (MFG)	Frontal pole (FP)
Orbitofrontal cortex (OFC)	Superior frontal gyrus (SFG)	Caudate nucleus (CAN)
Precuneus (PRE)	Postcentral gyrus (POG)	Putamen (PUT)
Anterior cingulate gyrus (aCG)	Precentral gyrus (PRG)	Thalamus (THL)
Posterior cingulate gyrus (pCG)	Anterior middle temporal gyrus (aMTG)	
Inferior frontal gyrus “pars opercularis” (IFGpo)	Posterior middle temporal gyrus (pMTG)	
